# Metabolic Energy-Based Modelling Explains Product Yielding in Anaerobic Mixed Culture Fermentations

**DOI:** 10.1371/journal.pone.0126739

**Published:** 2015-05-18

**Authors:** Rebeca González-Cabaleiro, Juan M. Lema, Jorge Rodríguez

**Affiliations:** 1 Department of Chemical Engineering, Institute of Technology, University of Santiago de Compostela, 15782, Santiago de Compostela, Galicia, Spain; 2 Institute Centre for Water and Environment (iWater), Department of Chemical and Environmental Engineering (CEE), Masdar Institute of Science and Technology, PO Box 54224, Abu Dhabi, United Arab Emirates; Virginia Tech, UNITED STATES

## Abstract

The fermentation of glucose using microbial mixed cultures is of great interest given its potential to convert wastes into valuable products at low cost, however, the difficulties associated with the control of the process still pose important challenges for its industrial implementation. A deeper understanding of the fermentation process involving metabolic and biochemical principles is very necessary to overcome these difficulties. In this work a novel metabolic energy based model is presented that accurately predicts for the first time the experimentally observed changes in product spectrum with pH. The model predicts the observed shift towards formate production at high pH, accompanied with ethanol and acetate production. Acetate (accompanied with a more reduced product) and butyrate are predicted main products at low pH. The production of propionate between pH 6 and 8 is also predicted. These results are mechanistically explained for the first time considering the impact that variable proton motive potential and active transport energy costs have in terms of energy harvest over different products yielding. The model results, in line with numerous reported experiments, validate the mechanistic and bioenergetics hypotheses that fermentative mixed cultures products yielding appears to be controlled by the principle of maximum energy harvest and the necessity of balancing the redox equivalents in absence of external electron acceptors.

## Introduction

Carbohydrates anaerobic fermentation towards volatile fatty acids (VFAs) has an increased interest due to its potential to provide building blocks from wastes towards a plethora of diverse valuable products. These chemical building blocks and complex biofuels [1, 2] or bioplastics [3–5] can be obtained from this system accompanied with short biomass production and low operational cost [6–8]. But, despite of its potential interest, significant improvement in the process control is needed towards an important boost of its industrial implementation, since products yielding highly varies with the substrate, inoculum and operational conditions [9, 10] with no clarified mechanistic interpretations [11–13].

Fermentations are environments with low energy available where microorganisms behave as highly efficient energy scavengers [14–22]. Mixed culture fermentation (MCF) presents high diversity in catabolic activities which increases the microbial population flexibility, facilitating the overall population survival by maximizing the energy harvest, and confronting successfully environmental changes that energetically constrain the microbial growth.

Previous modelling efforts in literature have attempted to describe product formation in MCF under different pH but have only partly succeeded or simply fell short [23–27]. Since modelling approaches based on constant fermentation reaction stoichiometry are not suitable to accurately describe the changing dependence of product yields with operational conditions [26, 28–30], a number of variable stoichiometry models were proposed [31–34]. These models however only achieved limited predictive and explanatory capacity in modelling the observed product shifts as function of changes in the operational conditions [11, 35–37]. This, together with the consideration that reproducible experimental products spectra have been obtained under similar operational conditions independently of the microbial inoculum [11], directly supports the hypothesis that biochemical and/or bioenergetics mechanisms play a key role on the observed product yields in MCF.

Energy-based modelling approaches have been proposed to mechanistically describe the impact of environmental conditions on MCF catabolic activities by means of bioenergetics [38, 39]. These recent models based on bioenergetics considerations did for the first time offer mechanistic insight on the possible reasons for the specific product yields observed. But did fall short in accurately predict the experimental product formation yields as function of operational conditions beyond very small ranges [24, 27], arising from the incompletely defined roles of electron carriers, the use of incomplete metabolic networks and the specific modelling approaches used for the transport processes across the cell membrane. We have identified these three aspects as the key factors limiting the predictive capacity of the existing energy-based MCF models which have to be addressed.

In this work, the model developed is applied to the understand of the pH role as operational variable into the product spectrum and this is targeted referring to the most complete experimental work done by Temudo et al. 2007 [11], previously performed by Zoetemeyer et al. 1982 [35], Horiuchi et al. 2002 [36] and Fang and Liu 2002 [37] which obtained similar results. The model presented in this study is the first able to accurately describe the pH effect on product formation in MCF and this is accomplished by directly addressing the above mentioned limitations of previous models.

## Model Description

### Metabolic network and transport

**Fig 1:**
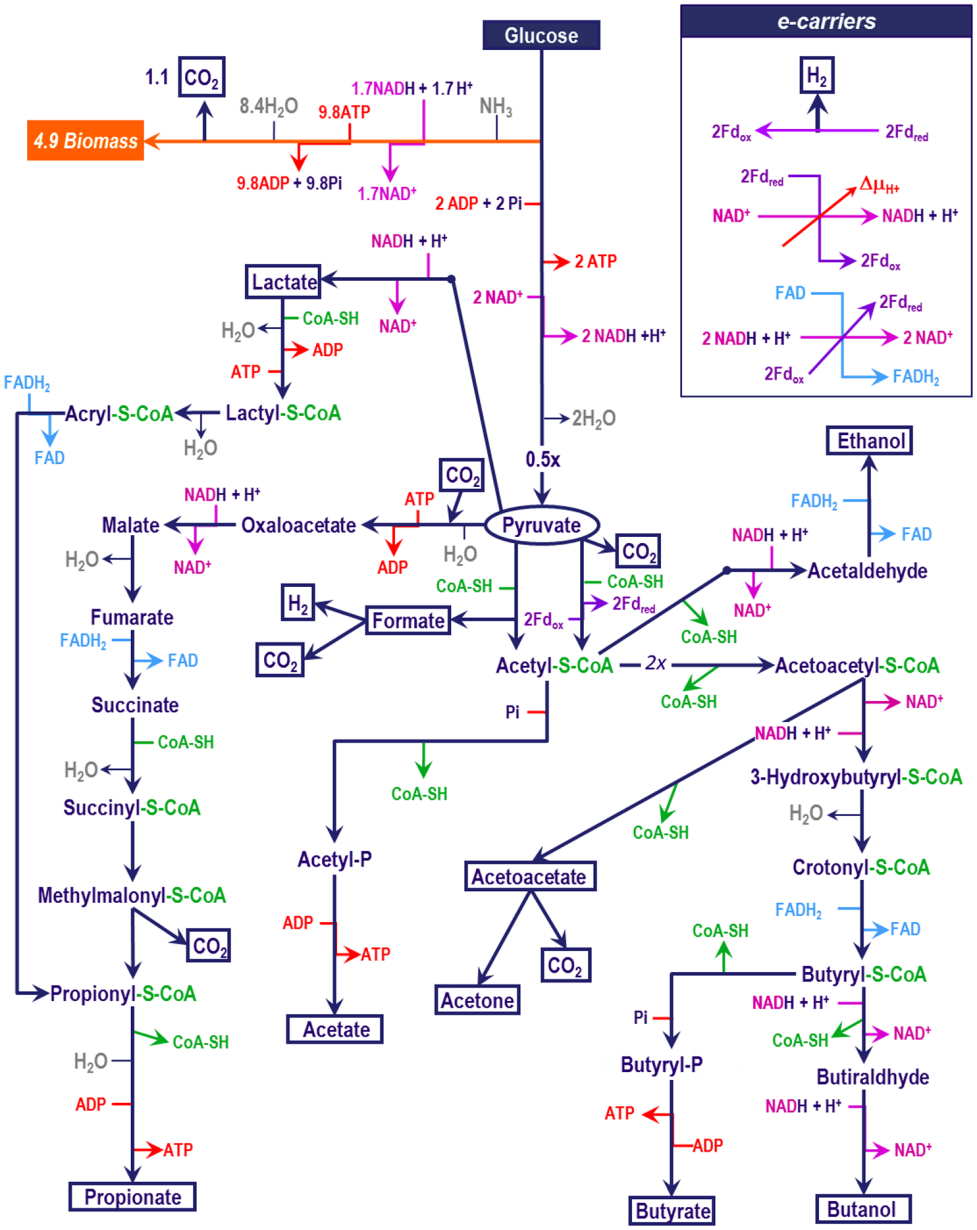
Metabolic reaction network model for the mixed culture fermentation of glucose.

The main goal of the model is to mechanistically describe the product spectrum shifts and trends experimentally observed in the mixed culture fermentation as function of the environmental conditions fixed in a continuous stirred tank reactor (Section C in S1 File and Section I in S1 File). To achieve this, the model proposed is based on the consideration of only one single hypothetical microbial population capable of performing all of the most important known metabolic fermentation pathways from glucose. This is in line with a similar approach previously proposed [24] neglecting microbial speciation or diversity at this stage. The network of metabolic fermentation reactions used (presented in Fig 1) was selected based on widely accepted literature [17, 25, 40–45] to include the most important and well described pathways towards the major fermentation products typically observed from glucose glycolysis.

The concentrations of chemical components in both the intra—(cytoplasmic) and extra—cellular volume domains, including pH and full ionic speciation are modelled and simulated dynamically (Section C in S1 File and Section E in S1 File). After analysing that no important kinetic bottlenecks seems to be present in the enzymatic reactions of the metabolic network (using an analogous method than in [21]) from all the intracellular chemical components shown in Fig 1, only those presented in Figure A in S1 File have been dynamically modelled with intermediate metabolites concentrations assumed almost constant. As it is known to occur in living cells, a control system was established to maintain intracellular concentrations and pH within valid homeostasis values for the microorganism [46–48] (further details can be found in Section G in S1 File). The model describes carefully the close tied up between bioenergetics [49] and all solute transport across the membrane in line with the chemiosmotic theory.

The optimization strategies widely applied in flux based analysis (FBA) methods for large metabolic networks [50], do typically use experimentally measured intracellular metabolite concentrations and optimize the metabolic network towards an objective such that the mass fluxes are predicted. The scope of this work differs from the conventional FBA in that it focuses on describing the microbial ecosystem reaction network interlinked with its environmental reactor conditions, such that a feedback of the reactor conditions impacts and is impacted by the microbial activity. Feedback from the environmental conditions into the network is modelled through mechanisms including transport energetics and maintenance requirements.

### Kinetic bottlenecks in the metabolic network

Feasible reaction intermediate intracellular metabolites concentrations can be assumed to have to fall between a maximum of 10 mM and a minimum of 1 μM [48] for physiological and kinetic reasons respectively. Then, kinetic limitations can be induced by thermodynamics [21, 51] implying unfeasible metabolite concentrations that could bring the pathway to a hold [21]. This is the case when extremely low product concentrations (< 1 μM, kinetically impossible to consume), or too high substrate concentrations (e.g. > 10 mM incompatible with cell homeostasis) [46, 47] are needed for an intermediate reaction to thermodynamically proceed (ΔG < 0).

**Fig 2:**
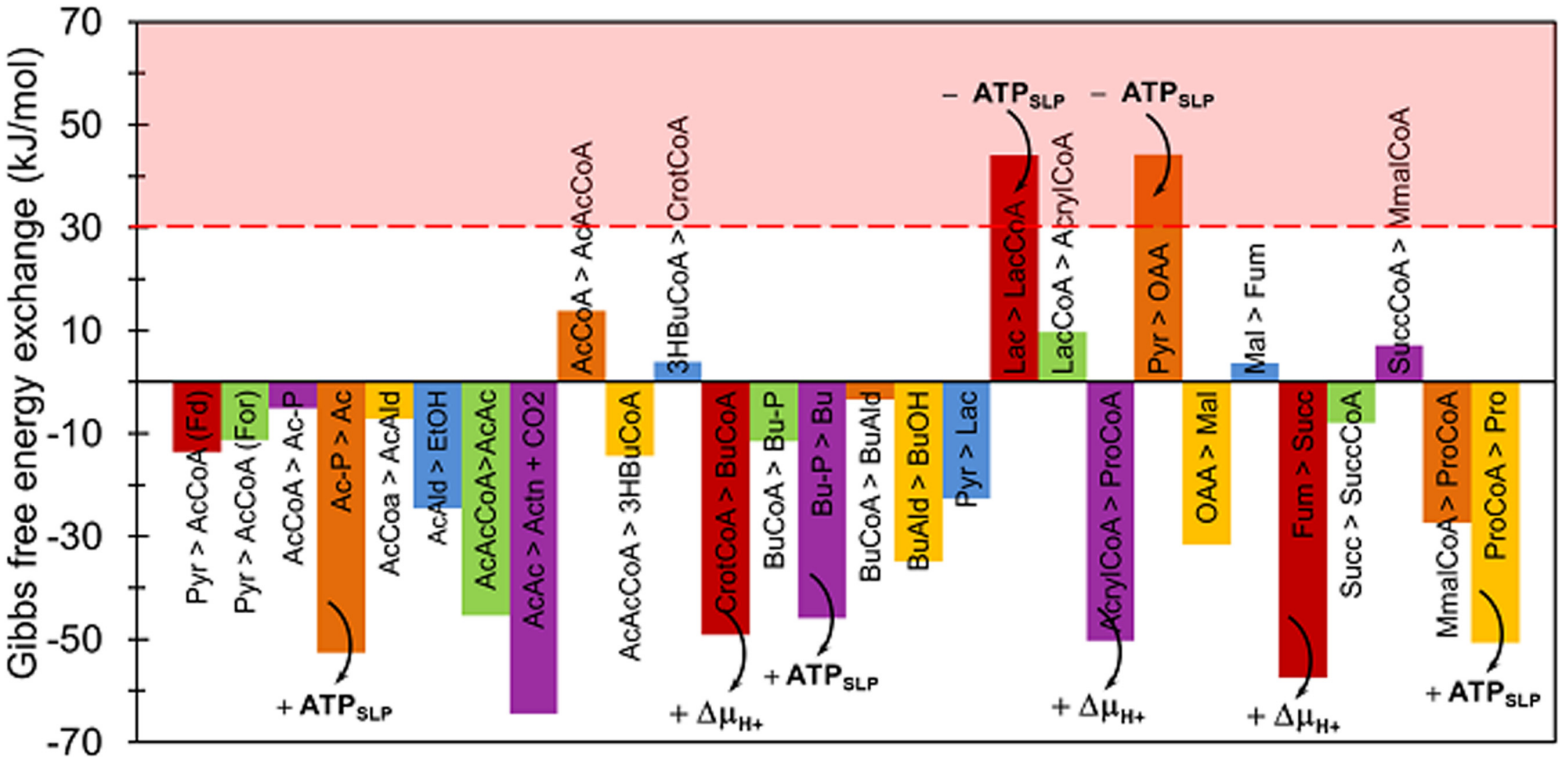
Gibbs free energy of central catabolic reactions of the metabolic network considered at 298.15 K, 1 atm, pH 7 and 1mM concentrations.

By conducting a thermodynamic assessment of the metabolic network presented in Fig 1, unavoidable energy limitations are not observed as energy demands of lower than 30 kJ are overcome by reasonable intracellular concentrations [48]. In Fig 2, it is detailed that the two reactions with Gibbs energy higher than 30 kJ are fuelled by an ATP hydrolysis.

### Metabolic energy production

The metabolism of a microbial cell can be described as a system organized in an energy harvesting catabolism coupled to an energy consuming anabolism and maintenance [52]. Cells can harvest catabolic energy through two mechanisms namely substrate level phosphorylation (SLP) and membrane related ion/proton translocations [53]. Both mechanisms end up yielding net ATP production, which is later used in anabolism and maintenance. The reaction sites in the fermentation network at which ATP via SLP is produced are well—known and defined (see Fig 1). However, energy can be harvested through proton extrusion across the membrane coupled to a less defined group of (or in principle to any) highly exergonic reactions.

The reversible nature of the ATP—synthase mechanism [54], allows for the contrary also to occur, fuelling an endergonic reaction coupled with the energy yielding intrusion of a proton (previously extruded concomitantly to ATP consumption). In such cases, energy might be spent by the cell to e.g. avoid a limitation in a specific catabolic pathway of interest [21].

Proton motive force (Δμ_H+_) is commonly defined as energy liberated when a proton enters in the cytoplasm [44] and that energy depends on the membrane potential and on the difference between the concentrations of the solutions separated by the membrane (Eq (1)).

$$\Delta \mu_{{\text{H}}^{+}}=\text{ F}\Delta \psi +\;\mathrm{RT}\cdot \ln\;10^{-\mathrm{pHin}}/10^{-\mathrm{pHout}}\left(\mathrm{kJ}/\mathrm{mo}{\text{l}}_{\text{e}-}\right)$$ [1]

Where Δψ is defined as Δψ = Δψ_in_ − Δψ_out_, with the inner membrane surface considered negative. Δψ has been reported near to a constant value depending on the microorganism and growth conditions [44]; in this model is considered constant at 0.2 V (Table B in S1 File). Any process increasing Δψ is assumed to be automatically compensated by an ATP coupled decreasing one [44, 54]. This, together with the intracellular pH assumed constant at 7 (Section G in S1 File), any Δμ_H+_ variations are solely considered due to variations on the external pH [55].

### Types and role of electron carriers

**Fig 3:**
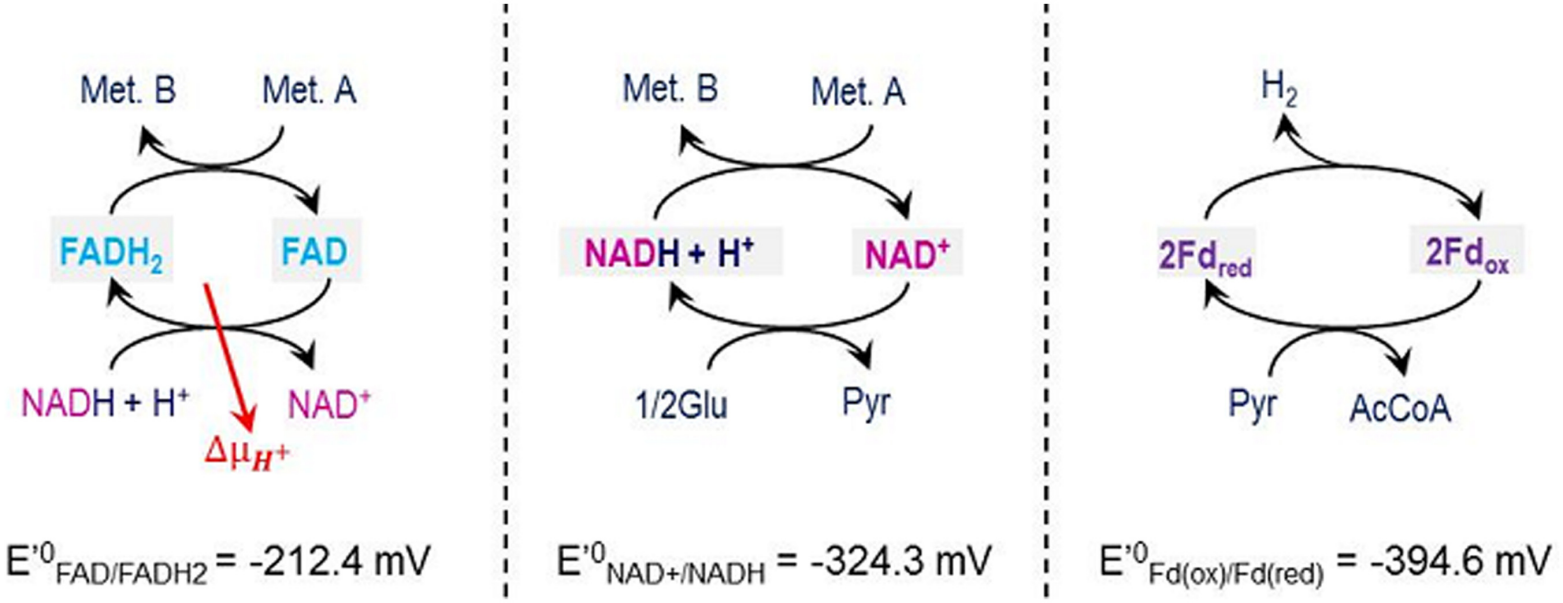
Electron carriers as included in the model with their specific reductive potential.

The availability of different electron carriers with different reductive potentials in the cell increases the energy harvest efficiency from catabolism as the most suitable carrier that can be coupled to the specific reaction according to its redox potential. This has been accounted for in the model and three electron carriers have been considered (Fig 3, the potential of each electron carrier is presented according to [56].

Ferredoxin (Fd_(ox)/(red)_), due to its more negative reduction potential, is the only electron carrier considered capable to direct H_2_ production [25, 32]. Ferredoxin reduction takes place only at pyruvate oxidation to acetyl—CoA (Fig 1) and it is assumed simultaneously to its oxidation that yields H_2_ (Fig 3) and which is consistent with experimental observations [11].

NAD(H) has a less negative reductive potential than ferredoxin and although in previous models NAD(H) was considered an electron carrier capable of direct H_2_ production [27], both experimental observations [11] and thermodynamic calculations [25, 32] (ΔG^01^ = +34.9 kJ/mol, for NADH oxidation towards soluble H_2_ production) strongly rule out direct H_2_ production from this carrier under cytoplasmic conditions [48]. NAD(H) takes part as electron carrier in specific metabolic reactions of the metabolic network (Fig 1) and it is balanced as a conserved moiety throughout the overall fermentation network stoichiometry. Although NADP(H) is also known to take part in some of the intracellular reactions, the small and not well defined differences between NADP(H) and NAD(H) and the difficulty to experimentally differentiate one from the other justify for the consideration of only NAD(H) in the model [57].

FAD(H_2_) is the third electron carrier considered in the model and it is associated to the highly exergonic metabolic reactions because of its less negative reduction potential. FAD reduction can be coupled to energy harvest by proton translocation [17]; thus, FAD(H_2_) electron carrier plays the role of an intermediate facilitating the coupling of a highly energetic metabolic reaction with the generation of proton motive force described with the oxidation of NADH following the mechanism presented in Eqs (2–4) [58–61] (Section B in S1 File and Section J in S1 File).

$$\mathrm{Met}.\text{A }+\;\mathrm{FAD}{\text{H}}_{2}\to \;\mathrm{Met}.\text{B }+\;\mathrm{FAD}$$ [2]

$$\mathrm{FAD}+\;2\mathrm{NADH}\;+\;2{\text{H}}^{+}+\;2\text{F}{\text{d}}_{\mathrm{ox}}\to \;\;\mathrm{FAD}{\text{H}}_{2}+\;2\mathrm{NA}{\text{D}}^{+}+\;2\text{F}{\text{d}}_{\mathrm{red}}$$ [3]

$$2\;\mathrm{Fdred}\;+\;\mathrm{NA}{\text{D}}^{+}\to \;2\;\mathrm{Fdox}\;+\;\mathrm{NADH}\;+\;{\text{H}}^{+}+\Delta \mu_{{\text{H}}^{+}}$$ [4]

Although Seedorf et al. 2008 [58] also suggested the direct production of H_2_ instead of one proton translocation in these highly energetic reactions, this was not considered due to the experimental observed 1:1 stoichiometry of H_2_ respect to pyruvate oxidation [11]. The same applies to discard the possibility of NAD reduction coupled to the pyruvate oxidation to acetyl—CoA.

In the network of Fig 1 we have included FADH_2_ associated to a proton translocation mediating the reduction of acetaldehyde to ethanol. This has not been reported but it has been found as a necessary mechanism to ensure the prediction of the observed high yield of ethanol production. In the ethanol pathway, the reduction of acetaldehyde to ethanol is typically exergonic (Fig 2), but the energy available is not as high as in other reductions associated to FADH_2_ oxidation. Then, in certain conditions, the available energy is not sufficient to yield a proton translocation (Δμ_H+_). However, reduction of ferredoxin by acetaldehyde has been reported [11, 62] thus, a similar electron bifurcation mechanism with FADH_2_ involved is proposed as possible in the ethanol pathway at conditions of low Δμ_H+_.

### Kinetic model of the metabolic reactions

Kinetic differences in metabolic reaction rates are expected to be not highly significant due to the nature of the system. The overall process rate is assumed controlled by the glucose uptake rate and glycolysis, which being highly exergonic, is never limited by thermodynamics. Therefore highly general and similar kinetic parameters are assumed across the board for all metabolic routes as they did not impact the predicted product spectrum trends during on our preliminary assessment (not shown). The overall process rate is modelled assuming one general maximum corresponding to 3 mol_e−_/mol_Cx_·h transferred in the glycolysis [52]. Any kinetic limitation due to glucose scarcity is modelled trough a Monod—like term.

$${\text{q}}_{\mathrm{SGly}}=\;{\text{q}}_{\text{S}}{}^{\max}\cdot \;\frac{{\text{S}}_{\mathrm{Glu}}}{{\text{K}}_{\text{s}}+{\text{S}}_{\mathrm{Glu}}}$$ [5]

where q_S_^max^ = 0.75 mol_Glu_/mol_Cx_·h is based on 4 electrons transferred in the glycolysis [43] (2 mol of NAD^+^ reduced per mol of glucose). No accumulation of intermediate metabolites is considered and a constant concentration of 7.5 mM assumed for intracellular pyruvate [63] (Section B in S1 File). All subsequent reactions in the network from pyruvate are only limited by glycolysis rate and their thermodynamic feasibility (Section D in S1 File and Section F in S1 File).

### Transport model for solutes across the membrane

Semi—permeable cell membranes in bacteria are known to allow for both the passive and active transport of solutes. Considering the model focus on product prediction, active transport of substrates (i.e. glucose) is not described and only products transport is modelled with distinction between uncharged species (free diffusion) and charged (active transport).

Passive transport is modelled considering that the lipid bilayer membrane controls only the cross of charged species but does not act as a barrier for small uncharged species. These species are assumed as freely diffusing through the membrane with no energy coupling or control from the cell homeostasis. A major difficulty arises from the largely unknown diffusion coefficients of all species through a cell membrane. If these values are accepted to be within the same order of magnitude, the main differences in diffusion rates come due to differences in the partition coefficients [27] (i.e. acid—base ionic speciation due to different intra and extracellular pH), which are thoroughly described by the model and then, the same diffusion coefficients for all uncharged species have been assumed (Section H in S1 File).

**Fig 4:**
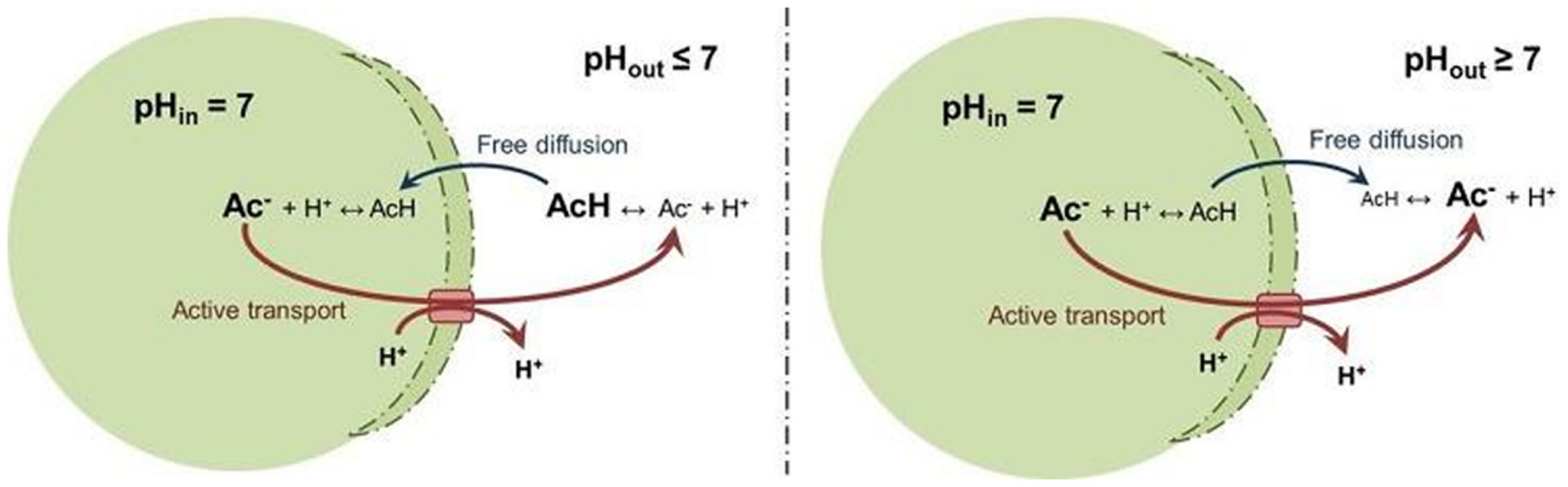
Transport model of acidic components across the membrane.

Active transport of charged and/or large molecules that are not freely diffusing through the cell membrane is coupled to metabolic energy exchange which is known to be performed by a diversity of transport proteins (ports) allowing only specific molecules to cross [44]. For the energy exchange, these ports are typically coupled to proton translocations, allowing endergonic transports to be fuelled by proton motive force if needed [64]. According to this, the transport of acidic components is modelled as active for their ionized deprotonated species and passive for their uncharged fully protonated species (Fig 4). In this scenario, the free diffusion passive transport term of a product extrusion might follow the same or opposite as active transport and therefore decreases or increases the active transport energy cost respectively.

Any changes in membrane potential associated to the transport of charged species are compensated by the needed proton translocation coupled to ATP hydrolysis. In this way the cell is capable of the maintenance of its homeostasis and membrane potential. The potential associated to a solute transport across the membrane is calculated by Eq (6) where z_i_ is the solute charge.

$$\;\Delta \mu_{\mathrm{Si}}=\;{\text{z}}_{\text{i}}\cdot \text{F}\Delta \Psi +\;\mathrm{RT}\cdot \ln\;\left[{\text{S}}_{\text{i},\mathrm{in}}\right]/\left[{\text{S}}_{\text{i},\mathrm{out}}\right]$$ [6]

All charged products considered in the fermentation model are negatively charged and their transport outside the cytoplasm leads to a decrease in the potential of the membrane (i.e. first term of (Eq (6)) [44]. An equivalent number of protons must be transported to maintain the membrane potential as well as the internal pH (Fig 4). Moreover, if the solute is transported against or in favour its chemical gradient across the membrane (i.e. the second term in Eq (6)), this energy might generate or consume additional proton motive force.

Overall, the number of protons (y) needed for transport fuelling (−) or harvested from transport energy surplus (+) are calculated as proposed in the well-known recycling model [65], Eq (7) (Section J in S1 File).

$$\text{y}=-\frac{{\text{z}}_{\text{i}}\text{F}\cdot \Delta \text{ψ }+\text{ RT}\cdot \text{ln}\frac{\left[{\text{S}}_{\text{i},\mathrm{in}}\right]}{\left[{\text{S}}_{\text{i},\mathrm{out}}\right]}\;}{\Delta \mu_{{\text{H}}^{+}}}+{\text{z}}_{\text{i}}$$ [7]

### Anabolism and decay

Anabolism and decay processes are modelled trough two overall mass balanced reactions. Anabolism (from glucose) is modelled limited by substrate and energy availability that comes from catabolism [66–68]. To account for the substrate limitation, Monod kinetic terms are used analogously than for glycolysis (Eq (5)) while for the energy availability the total amount of net ATP generated by catabolism is calculated.

A ratio of (ADP·Pi)/ATP concentrations is considered maintained constant at ΔG of −50 kJ/mol for ATP hydrolysis to preserve the cell homeostasis [69]. Part of the overall net ATP generated is allocated to a constant term of maintenance [70] (assumed 4.5 kJ/mol_Cx_·h [71] plus the maintenance associated to the active transport for pH homeostasis [44] (Section K in S1 File)). When concentrations of ATP increase the energy liberated in its hydrolysis to more than 50 kJ/mol, anabolism proceeds, if the contrary, the population decays.

### Selection of dominant metabolic pathways

The fundamental hypothesis in the model to describe product formation is that the dominant catabolic pathways will be those that return more net energy as ATP and consequently lead to the highest biomass growth. This is estimated by lineal optimization of the yield for each metabolic branch of the network (Fig 1) per mol of pyruvate produced (χ_i_) to maximize ATP yielding in catabolism (Section L in S1 File). The optimization is constrained by closing electron balances (as NADH equivalents, Section B in S1 File) and a zero or net HCO_3_^−^ production as it is not fed and it acts as substrate in some catabolic reactions (Fig 1).

$$\chi_{\text{i}}=\mathrm{lin}\_\mathrm{opt}(\text{d}\left[\mathrm{NADH}\right]/\mathrm{dt}=\;0;\text{d}\left[\mathrm{HC}{\text{O}}_{3}{}^{-}\right]/\mathrm{dt}\geq \;0;\;0\;\leq {\text{c}}_{\text{i}}\leq \;1)$$ [8]

By closing the mass balances all pyruvate produced by glycolysis is consumed by the metabolic pathways selected (such that $\sum_{\text{i}=1}^{\text{i}=\text{N}}\chi_{\text{i}}=1$) (Section L in S1 File).

## Results

Model simulations were conducted until steady state for an HRT of 8 hours and for pH values between 4 and 8.5 with a resolution of 0.5 pH units. The same carbon source as in Temudo et al. 2007 [11], 4 g/L of glucose, was used and ammonium was fed in non—limiting concentrations (Section M in S1 File).

**Fig 5:**
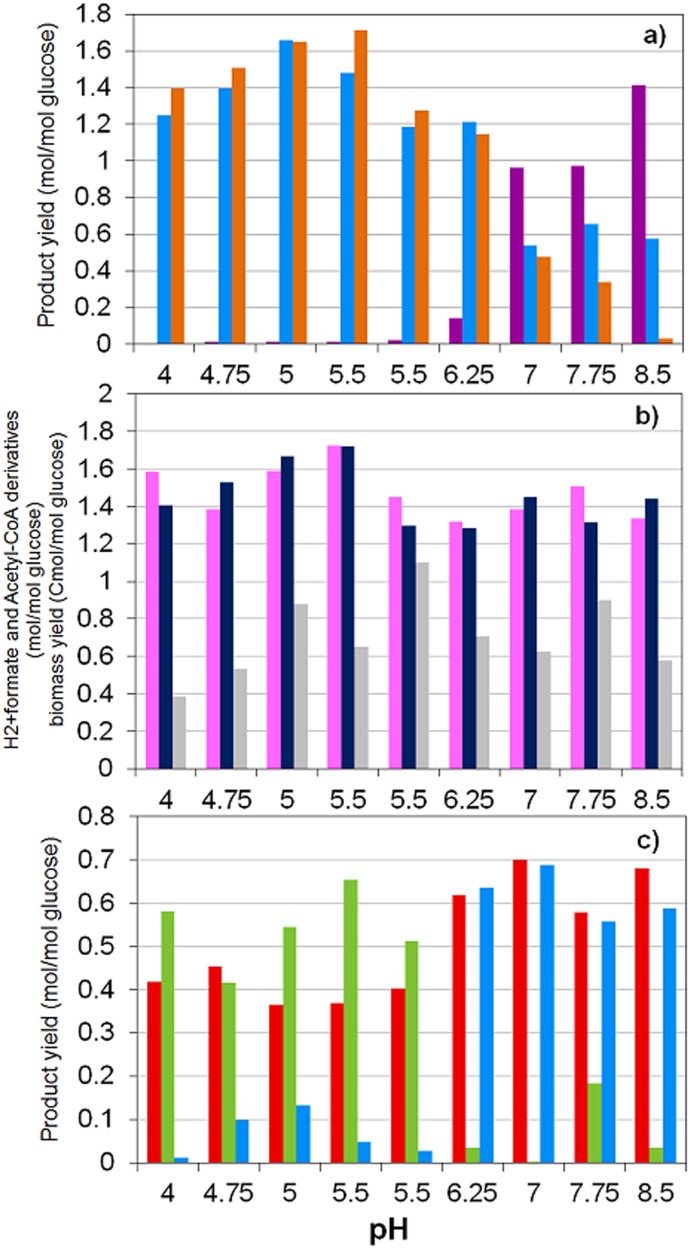
Experimental steady state yields as reported by Temudo et al. 2007 [11] (at 20 hours of HRT for pH ≤ 5.5 and at 8 hours of HRT for pH ≥ 5.5). **a)** Influence of pH on formate, CO_2_, and H_2_ yields per glucose consumed by MCF. Yields of: (*purple*) formate, (*light blue*) CO_2_ (gas and dissolved CO_2_ + HCO_3_^−^) and (*orange*) H_2_. **b)** Influence of pH on acetyl—CoA derivatives, H_2_ + formate and biomass yields per glucose consumed by MCF: (*pink*) Total acetyl—CoA derivatives as sum of acetate, butyrate (x2) and ethanol yields; (*navy blue*) sum of H_2_ and formate yields and (*grey*) biomass yield. **c)** Influence of pH on dissolved products yields per glucose consumed: (*red*) acetate; (*purple*) propionate; (*light green*) butyrate and (*cyan*) ethanol.

The observations by Temudo et al. 2007 [11] are to our knowledge the only experimental work reporting closed electron and carbon balances (Fig 5). In this section, a comparison between these experimental results and the outputs of our model is presented for the major products observed in glucose MCF (for the results of minor products see S2 File).

### Formate vs H_2_

**Fig 6:**
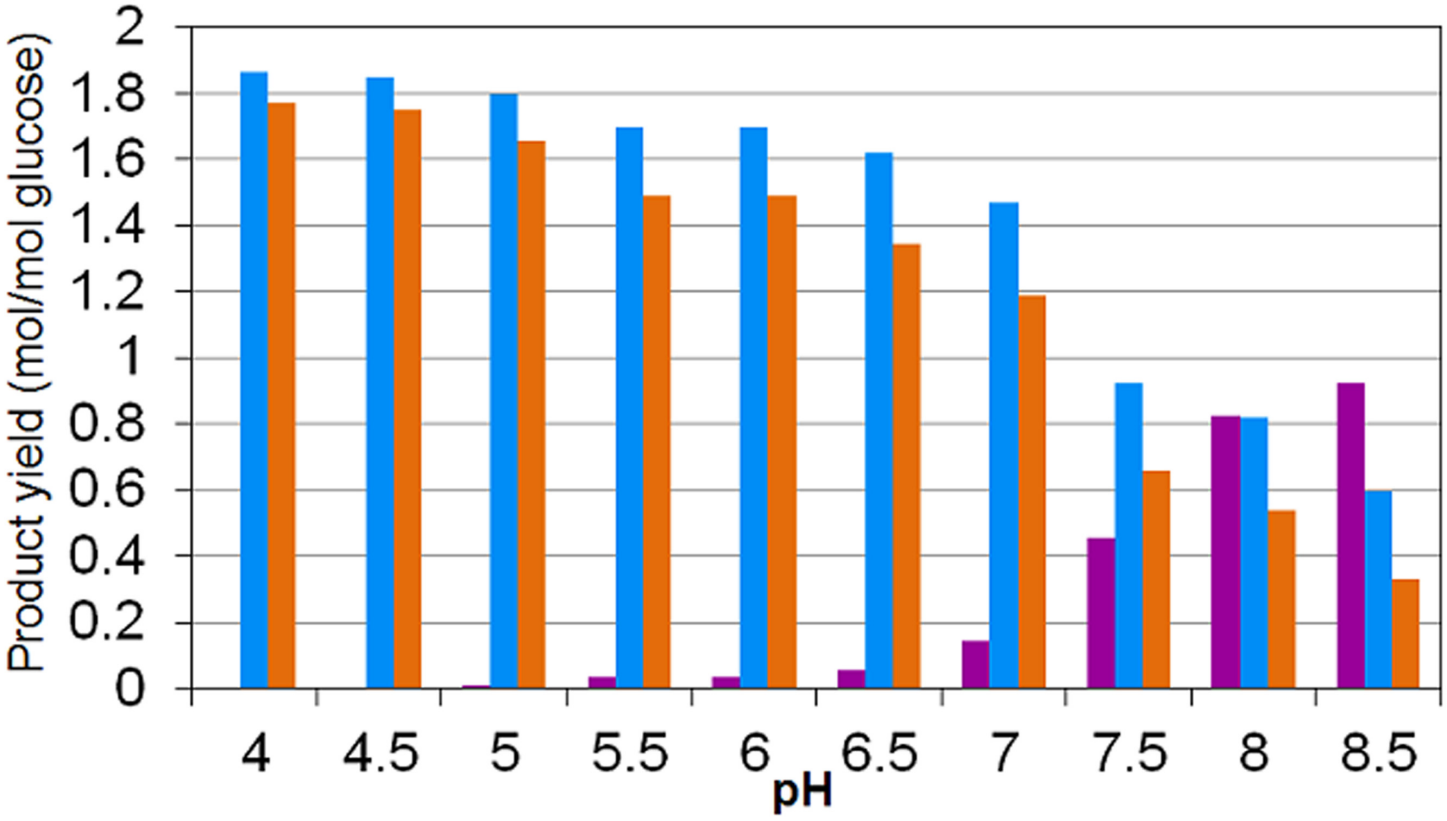
Model simulation results on the influence of pH on formate CO_2_, and H_2_ yields per glucose consumed. Yields of: (*purple*) formate; (*light blue*) CO_2_ (gas and dissolved CO_2_ + HCO_3_^−^) and (*orange*) H_2_. (Compared to the experimental results presented in Fig 5a).

**Fig 7:**
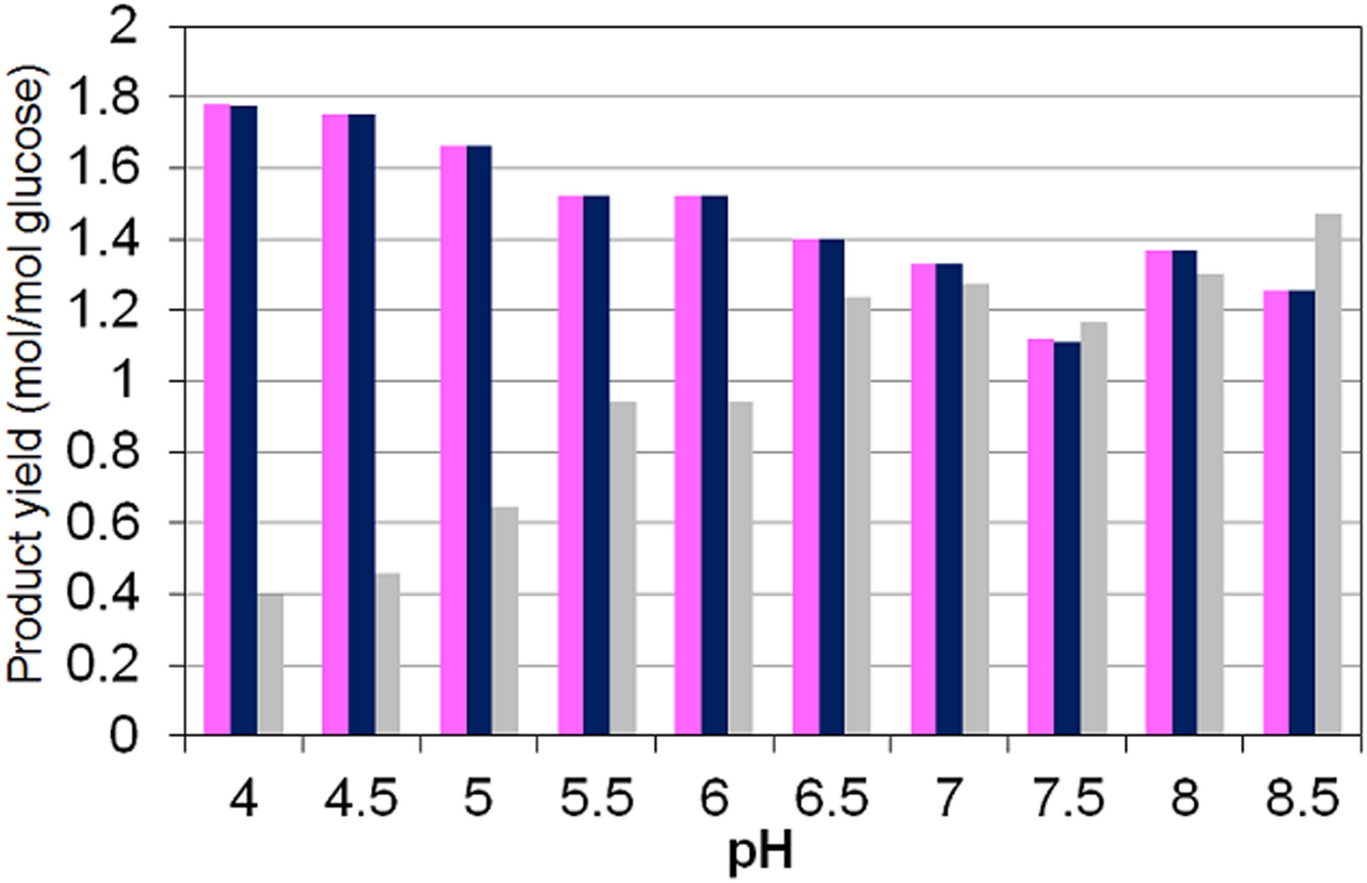
Model simulation results on the influence of pH on acetyl-CoA derivatives, H_2_ plus formate and biomass yields per glucose consumed. Yields of: (*pink*) Total acetyl—CoA derivatives as sum of acetate, acetoacetate, butyrate (x2), butanol (x2), butyraldehide (x2), ethanol and acetaldehyde yields; (*navy blue*) sum of H_2_ and formate yields and (*grey*) biomass yield. (Compared to the experimental results presented in Fig 5b).

The experimental observations reported [11] indicate that H_2_ and CO_2_ are predominant at low pH while formate production dominates at higher pH (Fig 5). The sum of H_2_ and formate yields returning approximately the same as the sum of acetyl—CoA products serves as partial validation of the metabolic network assumed for the model (Fig 1). The model simulations results succeed in predicting these yield trends as experimentally observed (Figs 5, 6 and 7).

The energy yields during pyruvate oxidation to acetyl—CoA between H_2_ production (through ferredoxin) and formate production (through ferredoxin or direct pyruvate oxidation) are equivalent and no evidence suggests that extracellular pH could have an impact on them [11]. However, the model is successful at predicting the shift from CO_2_ + H_2_ to formate at high pH (Fig 6) being therefore attributed to the extra energy harvested through formate active transport across the membrane at high pH together with the increased energy costs of transporting HCO_3_^−^ outside the cytoplasm due to higher inorganic carbon solubility when pH increases.

Differences between model predicted higher biomass yields at high pH (Fig 7) and experimental observations cannot be clearly attributed. The reduction of HRT at higher pH in the experimental operation by Temudo et al. 2007 [11] (Fig 5b) with the associated change in growth rate could be a cause of discrepancy that is not fully described in our model however, they also observed a reduction of the biomass yield referred to the ATP harvested when the external pH is low and the fermentation is yielding butyrate at high concentrations [10, 72]. Alternatively, also variations of the internal pH (physiologically feasible between 5.5 to 7 [73]) which are not considered in the model (internal pH is fixed at 7) could account for significant different maintenance energy costs and efficiencies impacting biomass yield values.

### Product spectrum

**Fig 8:**
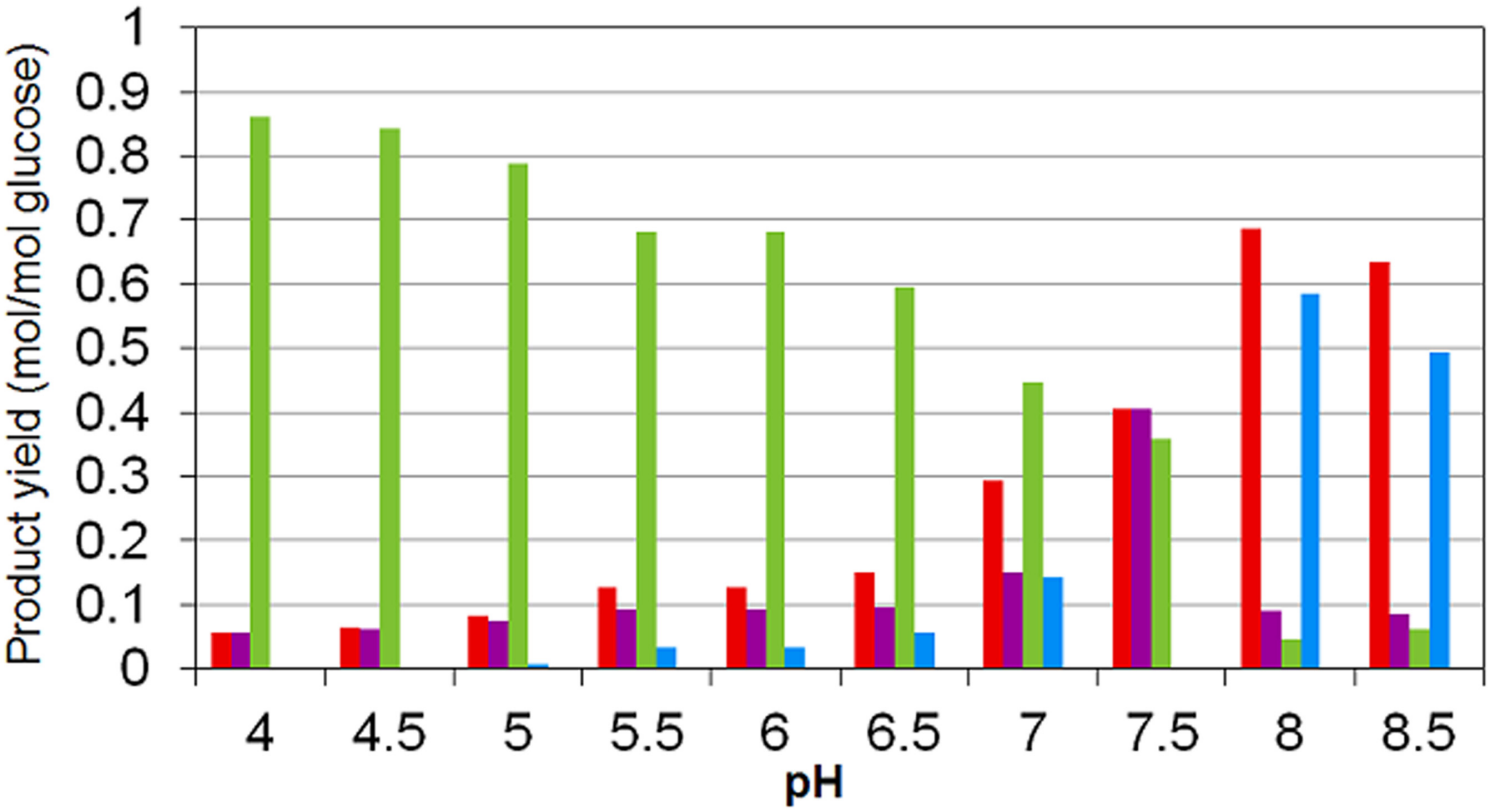
Model simulation results on the influence of pH on dissolved products yields per glucose consumed by MCF. Yields of: (*red*) acetate; (*purple*) propionate; (*light green*) butyrate and (*cyan*) ethanol (Compared to the experimental results presented in Fig 5c).

In Fig 8 the organic products spectrum predicted by the model is presented. As experimentally observed (Fig 5c), acetate and butyrate high concentrations are predicted at low pH whereas a shift from butyrate to ethanol at higher pH is observed.

Fermentation with acetate as only product has not been experimentally reported being its production always accompanied by other more reduced products yielding [74]. The main reason lies in the necessity to oxidize the NADH produced in glycolysis, which, in absence of external electron acceptors (such as O_2_, NO_3_^−^), leads to electrons allocation in reductive fermentation steps. The need for this complete electron balance fully accounted for in the model, is a main constraint during the maximization of ATP production and appears to be in line with the experimental observations.

At low pH, butyrate and acetate are predicted as major products as well as experimentally reported (Figs 5c and 8) with presence of other more reduced products. At high pH, butyrate production appears to dramatically diminish and ethanol and acetate are predicted as well as experimentally reported as the major products (Figs 5c and 8). This clear shift in products is successfully predicted by the model but at a higher pH than in the observations. The presence of propionate is predicted as secondary reduced product (Fig 8) associated to acetate production while is not present in the experimental observations by Temudo et al. 2007 [11] where ethanol or glycerol are found in higher concentrations (Fig 5c). However, propionate producers seems to have limited presence in the mixed culture inoculum used by Temudo et al. 2007 [11] because in contrary, it has been reported as a major product together with acetate at approximately the same pH than the predicted by the model in the experiments developed by other authors [36, 37].

Based on the metabolic network in Fig 1 acetate plus ethanol; acetate plus propionate; and butyrate production are the three combinations of catabolic products leading, closed electron balances, to a maximum energy harvest per unit of glucose consumed (one ATP by SLP formation plus the energy of one proton translocation per unit of glucose). These products are the ones with higher yields predicted by the model as well as experimentally reported [11, 36, 37].

Considering that Δμ_H+_ is modelled as only depending on external pH, when it is low, ΔpH increases and more energy is needed to translocate one proton across the cell (Δμ_H+_ increases). In ethanol pathway, the energy available during FADH_2_—mediated acetaldehyde reduction is not as high as that in crotonyl—CoA reduction (part of the butyrate pathway) or that in fumarate or in acryl—CoA reductions (part of the propionate pathways) (see Figs 1 and 2). Therefore, at low pH, no proton translocation is possible when ethanol is yield and butyrate is predicted as major product.

Butyrate production at low pH supposes the less retention of the concomitantly produced inorganic carbon, with its increased transfer as CO_2_ to the gas phase. At higher pH however, butyrate production energetic yield decreases as more total inorganic carbon is kept in solution. This favours the propionate pathway energetics, as it does not imply any decarboxylation. Then, at pH range between 6 and 7.5, acetate and propionate together with formate production increases (Figs 6 and 8).

Ethanol pathway at high external pH (8, 8.5) permits one proton translocation (Δμ_H+_ is low in these conditions) which elevates the capacity of energy harvest by this pathway making together with acetate yielding, its production competitive comparatively to yield butyrate or acetate plus propionate. This comes together with the higher energy available for acidic components transport at high pH, and makes the ethanol production more favourable than propionate in terms of energy yield as formate, which is the strongest acid, is produced in ethanol pathway (Fig 1).

### Limitations of the model and opportunities for development

The proposed model provides insights into energetically based mechanisms of the trends and shifts observed on the product spectrum in glucose fermentations; the model has some limitations and areas for further development to be considered including:

1. The numerical accuracy on the prediction of product yields is expected to improve if more kinetic information is incorporated. Currently no specific kinetic information has been modelled for each intracellular reaction as the dynamical description of each of the metabolic fluxes and transports was not targeted as a main objective, the steady state products yield were targeted instead controlled by energetic considerations.
2. Alternative additional fermentation products such as glycerol, currently not included, could potentially have roles in the balancing of electron equivalents (NADH) and could affect the model prediction capacity in some cases.
3. Alternative additional mechanisms of NADH/NAD^+^ recovery impacting the electron balances and possibly involving other electron carriers not considered could have significant roles [11].
4. The impact of anabolism and decay on the NADH balance has not been included and only the oxidation or reduction present in catabolism (as included in Fig 1) was considered. This possibly implies a slight underestimation of the yields of the more reduced products (since glucose has a slight lower degree of reduction than microbial biomass assumed as CH_1.8_O_0.5_N_0.2_).
5. All solutes have been modelled using the same diffusion coefficient through the cell membrane due to the lack of reliable values for many of them. Differences in these coefficients could slightly increase or reduce the predicted product yields under specific operational conditions.
6. Physiological characteristics not considered in the model, such as variable internal pH or variable membrane potential, could lead to changes in the energetics of specific products and therefore their predicted yields by the model.

The current modelling framework as presented, can be adapted to describe the fermentation of other substrates (e.g. xylose or glycerol) by modification of the upper parts of the metabolic network. The model application to the study of more complex substrates (constituted by lipids, carbohydrates or proteins) is however thought to be more troublesome due to the number of additional factors that could deviate from the model assumptions (these include inhibitions or physicochemical solubility—related factors). Experimentally, when using complex substrates, the control of the products spectrum is difficult, however, mechanistic insights on their fermentation, such as those obtained through in this study with glucose, do contribute towards the overall understanding and accelerate the industrial application of these bioprocesses towards valuable products recovery from wastes. Extensions of the metabolic network to address additional or more complex substrates are not expected to pose major impacts on computational times to solve the model as the already long simulation times required are mainly caused by short integration time steps due to the stiffness of specific variables.

## Conclusions

The model developed, based on the optimization of the ATP production under a detailed metabolic network and with a full account of the electron balances and membrane transport energetics is, to our knowledge, the first mechanistic model succeeding in the prediction of observed shifts in major fermentation products with external pH, including the shift between CO_2_/H_2_ and formate production.

The breakthrough improvement respect to previous models is attributed to the more comprehensive account for the different electron carriers and their roles, a more complete metabolic reaction network and a detailed modelling of the energetics of solutes transport across the cell membrane. Additional value comes from the minimum parameter fitting needed and the fact that all results obtained are mechanistically related.

Under this approach, mixed culture fermentations, known to take place under strong energy limitation, are treated as networks that optimize the energy harvest rate of the overall mixed microbial population. The model focuses on first principles and on the energetic constrains imposed by the environment and avoids other specific physiological and ecological mechanisms. The results obtained under this modelling approach strongly support the hypothesis that mixed culture microbial ecosystems can be described as highly efficient energy harvesters in which, independently from the microbial community composition, the conditions of maximum energy harvest rate are achieved in the long term.

## Supporting Information

S1 File (PDF)

S2 File (PDF)
