# Supplementary material for: Metabolic Energy-Based Modelling Explains Product Yielding in Anaerobic Mixed Culture Fermentations
Source: PLoS One. 2015 May 18;10(5):e0126739. doi: 10.1371/journal.pone.0126739 (PMC4436308; doi:10.1371/journal.pone.0126739)
Supplement: S1 File — (PDF) [file pone.0126739.s001.pdf]

# Supporting Information

## Metabolic energy-based modelling explains product yielding in anaerobic mixed culture fermentations

R. González-Cabaleiro[1], J. M. Lema[1] and J. Rodríguez[2]

[1]Department of Chemical Engineering, Institute of Technology.
University of Santiago de Compostela, 15782 Santiago de Compostela, Galicia, Spain.
[2]Institute Centre for Water and Environment (iWater) Department of Chemical and Environmental Engineering (CEE)
Masdar Institute of Science and Technology. PO Box 54224 Abu Dhabi, United Arab Emirates

# Model description

## A. Introduction

The energy-based model developed in this work describes the metabolic processes occurring in mixed culture fermentation (MCF) of glucose. The model proposed, assumes as fundamental hypothesis that the bacteria tend to maximize the energy harvested out of the surrounded system. This energy is later used for maintenance and growth. To maximize this energy, bacteria optimize its metabolic strategy yielding different products out of the available substrates in the system.

The model proposed studies the consumption in anaerobic conditions of an easily degradable substrate like glucose in a continuous stirred tank reactor. A source of nitrogen is added in the reactor and a constant pH is considered. Butyrate, propionate, acetate, ethanol, butanol, lactate, acetone, acetoacetate, butyraldehyde, acetaldehyde, oxalate, malate, succionate and fumarate are the fermentative products considered. A single microbial cell able to do all the fermentative metabolic pathways is assumed. Depending on the energy available in the system, the bacteria shift from one metabolic pathway to another. As decision criteria, the maximization of energy harvested for anabolism and maintenance per unit of time by the catabolic pathway is used. Because our model is built considering that all the energy harvested has its end point in the formation of new phosphate links (to later break them for maintenance and growth), the maximization of ATP production rate in the catabolic process is used as decision criteria between the catabolic pathways.

The model was built in a generalized form, in order to keep the possibility to include new chemical reactions open, and reduce the number of parameters used. Simplicity and proximity to the basic concepts are always maintained.

## B. The reaction network

The rates of the network branches are defined only dependent on the glucose availability. All the rates of all reactions in each catabolic branch are the same and the intermediate metabolites accumulation is assumed zero. Therefore, considering that the intermediate metabolites are produced and consumed at the same rate, we can neglect their dynamic description to avoid working with a big number of variables.

Ferredoxin is assumed directly re-oxidized after reduction in the conversion of pyruvate to acetyl-CoA producing $H_2$, thus oxidized and reduced forms concentrations of this electron carrier are considered as constants and neglected in the model. FAD($H_2$) concentrations can be as well eliminated from the network considering the eqns. 2—4 of the main manuscript, where the sum of the whole mechanism supposes the metabolite reduction plus the oxidation of one NADH and one proton translocation eqn. S1—S4.

$$\text{Met.A} + FADH_2 \rightarrow \text{Met.B} + FAD \qquad [\mathbf{S1}]$$

$$FAD + 2NADH + 2H^+ + 2Fd_{ox} \rightarrow FADH_2 + 2NAD^+ + 2Fd_{red} \qquad [\mathbf{S2}]$$

$$2\ \text{Fdred} + NAD^+ \rightarrow 2\ \text{Fdox} + NADH + H^+ + \Delta\mu_{H+} \qquad [\mathbf{S3}]$$

$$\textbf{Total:}\quad \text{Met.A} + NADH + H^+ \rightarrow \text{Met.B} + 2NAD^+ + \Delta\mu_{H+} \qquad [\mathbf{S4}]$$

Then, a simplified network is used in the model instead the one presented in Fig. 1 of the main manuscript, where only substrates and final products are considered, as well as ATP production via SLP and only NADH as electron carrier (Fig. A).

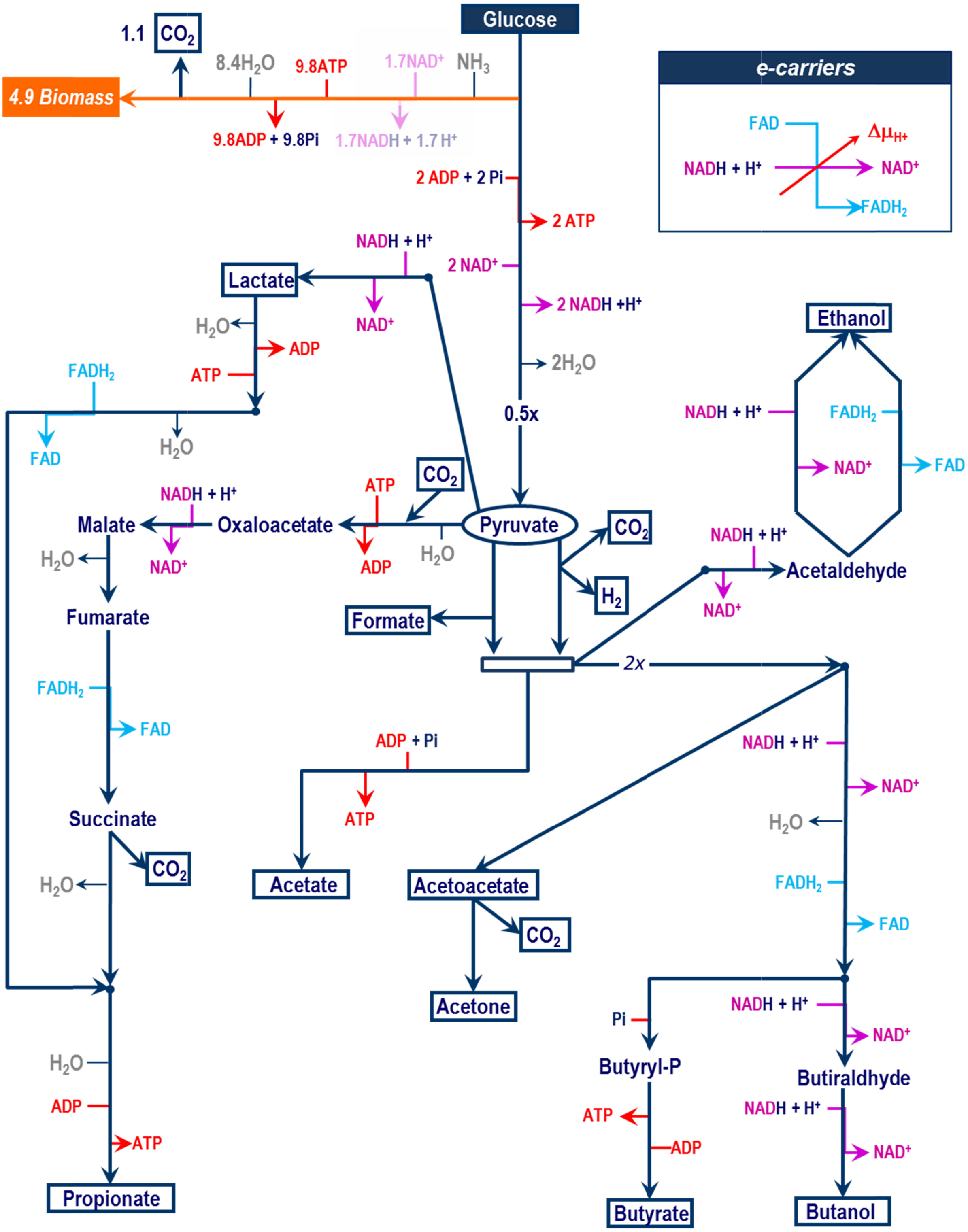


**Figure A** Simplified reaction network used for MCF model

# C. The dynamic reactions

The dynamic reactions of the model are the mass balances of each reactor compartment considered (Fig. B). Each compartment defines a volume: $V_r$ ($L_r$) the working volume, $V_{liq}$ ($L_{liq}$) the volume of liquid (working volume subtracting the volume occupied by the biomass), $V_x$ ($L_x$) the biomass volume, and $V_{gas}$ ($L_{gas}$) the head space volume.

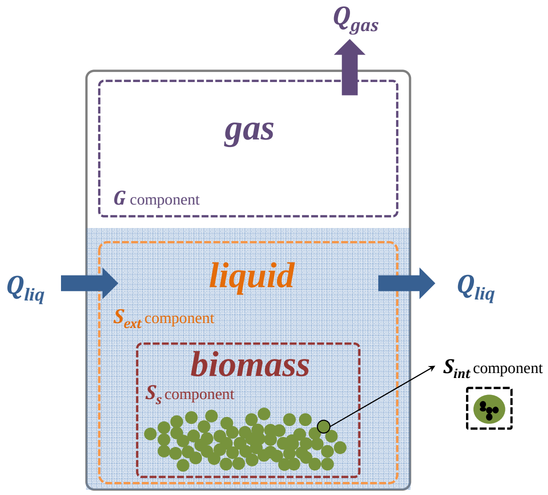


**Figure B** Scheme of the reactor sections considered.

Intracellular balance (no transportable components, $S_i$ $mol_{Si}/L_x$):

$$\frac{dS_i}{dt} = R_i \qquad \textbf{[S5]}$$

Intracellular balance (transportable components, $S_j$ $mol_{Sj}/L_x$):

$$\frac{dS_j}{dt} = R_j + R_{T,j} \qquad \textbf{[S6]}$$

Extracellular balance ($S_k$, $mol_{Sk}/L_{liq}$):

$$\frac{dS_k}{dt} = \frac{1}{V_{liq}} \cdot Q_{liq} \cdot (S_{k,inf} - S_k) + R_{T,k} \qquad [\mathbf{S7}]$$

Biomass balance (X, $mol_{Cx}/L_r$):

$$\frac{dX}{dt} = -\frac{X}{SRT} + R_X \qquad [\mathbf{S8}]$$

Gas balance (G, $mol_{Gl}/L_{gas}$):

$$\frac{dG_l}{dt} = -\frac{Q_{gas}}{V_{gas}} \cdot G_l + R_l \qquad [\mathbf{S9}]$$

The solid retention time (SRT) is assumed in all the simulations equal to the hydraulic retention time (HRT, Table A)

The states of the model are the total concentrations of chemical components accounting for all the liquid ionic species concentrations when applicable (e.g. equations S10 and S11).

$$[HAc]_{state} = [HAc] + [Ac^-] \qquad [\mathbf{S10}]$$

$$[IC]_{state} = [CO_2] + [H_2CO_3] + [HCO_3^-] + [CO_3^{2-}] \qquad [\mathbf{S11}]$$

In order to solve the states of the model for each step of time through the differential equations (eqns. S5—S9), it is necessary to accurately model the reactions and transport terms of intra- and extracellular concentrations.

## D. Thermodynamics of catabolic reactions

The $\Delta G$ of each metabolic reaction is calculated using a generalized routine. Knowing the stoichiometry of each reaction ($\nu_i$, is the stoichiometry coefficient for each component i of the reaction considered), actual $\Delta G$ at each step of simulation time is calculated from the $\Delta G^0$ values obtained from literature [1-5] that have been previously pre-stored in a matrix.

$$\Delta G = \Delta G^0 + R \cdot T \cdot \ln \prod_i a_i^{\nu_i} \qquad \textbf{[S12]}$$

Where R is the constant of gases 8.314 J/mol·K and T is the temperature considered 298.15 K for all the simulations.

The activity of every chemical component ($a_i$, eqn. S13) is calculated considering the ionic strength of the solution using the Debye-Hückel law, eqn. S14 [6], where $C_i$ is the concentration and $z_i$ is the charge of each component.

$$a_i = \gamma_i \cdot C_i \qquad \textbf{[S13]}$$

$$-\log(\gamma_i) = A \cdot z_i^2 \cdot \sqrt{I_C} \qquad I_C < 0.001\ M$$
$$-\log(\gamma_i) = \frac{A \cdot z_i^2 \cdot \sqrt{I_C}}{1 + B \cdot \sqrt{I_C}} \qquad I_C > 0.001\ M \qquad \textbf{[S14]}$$

The ionic strength is calculated according eqn. S15.

$$I_C = 0.5 \cdot \sum_{i=1}^{N} C_i \cdot z_i^2 \qquad \textbf{[S15]}$$

Depending on its $\Delta G$ the direction of the reaction is fixed considering a minimum energy necessary to run the reaction ($\Delta G_{min}$) of -2 kJ/mol. The factor f permits to calculate the direction of the reaction according to the available energy.

$$\begin{aligned} f &= 1 & \Delta G &< \Delta G_{min} \\ f &= 0 & \Delta G_{min} &< \Delta G < -\Delta G_{min} \\ f &= -1 & \Delta G &> -\Delta G_{min} \end{aligned} \qquad \textbf{[S16]}$$

## E. Acid-base equilibria

The acid-base equilibria are modelled algebraically [7].

$$[HAc] \xleftrightarrow{Ka_1} [Ac^-] + [H^+] \qquad Ka_1 = \frac{[Ac^-] \cdot [H^+]}{[HAc]} \qquad \textbf{[S17]}$$

All the equilibrium constants are calculated directly from the Gibbs energy of formation obtained in literature. The solution of the derivative equation returns the total concentration of a chemical component. A general routine is implemented to calculate from the total concentrations, all the chemical species consider in the model (eqn. S18 and eqn. S24).

$$\text{Not hydrated form activity: } [\text{Not Hyd.}] = K_d \cdot \frac{C_T \cdot \left[H^+\right]^N}{A} \quad \textbf{[S18]}$$

$$\text{Fully protonated form activity: } [\text{Hyd.}] = \frac{C_T \cdot \left[H^+\right]^N}{A} \quad \textbf{[S19]}$$

$$1^{st} \text{ deprotonated form activity: } \left[1^{st}\,\text{dep.}\right] = \frac{C_T \cdot Ka_1 \left[H^+\right]^{N-1}}{A} \quad \textbf{[S20]}$$

$$2^{nd} \text{ deprotonated form activity: } \left[2^{nd}\,\text{dep.}\right] = \frac{C_T \cdot Ka_1 \cdot Ka_2 \left[H^+\right]^{N-2}}{A} \quad \textbf{[S21]}$$

$$3^{er} \text{ deprotonated form activity: } \left[3^{rd}\,\text{dep.}\right] = \frac{C_T \cdot Ka_1 \cdot Ka_2 \cdot Ka_3 \left[H^+\right]^{N-3}}{A} \quad \textbf{[S22]}$$

$$k^{st} \text{ deprotonated form activity: } \left[k^{st}\text{dep.}\right] = \frac{C_T \cdot \prod_{j=1}^{j=k} Ka_j \left[H^+\right]^{N-j}}{A} \quad \textbf{[S23]}$$

$$A = (1+K_d)\cdot\left[H^+\right]^N + Ka_1\left[H^+\right]^{N-1} + Ka_1 \cdot Ka_2 \cdot \left[H^+\right]^{N-2} + Ka_1 \cdot Ka_2 \cdot Ka_3 \cdot \left[H^+\right]^{N-3} + \sum_{k=4}^{k=N}\left(\prod_{j=1}^{j=k} Ka_j \left[H^+\right]^{N-k}\right) \quad \textbf{[S24]}$$

pH is necessary to solve these equilibriums. In the extracellular solution, pH is fixed and supposed controlled, cations and anions are balanced in each simulation step maintaining the pH at a fixed value. For the cytoplasm, the pH is calculated according the species present in the solution. A numerical method like Newton-Raphson is necessary to calculate pH and species concentrations using as objective function the balance of charges, eqn. S25.

$$\sum_{i=1}^{N} C_i \cdot z_i = 0 \quad \textbf{[S25]}$$

For this routine, activity coefficients are neglected, because their impact in the pH calculation was demonstrated negligible [8].

# F. Reactions kinetics

All the catabolic reactions of the network are limited for the glycolysis rate. Assuming 3 e-mol/mol$_{Cx}\cdot$h as maximum yield, and limiting it with Monod-like terms accounting for glucose availability, the rate of glucose consumption is as eqn. S26.

$$q_{SGly} = q_S^{max} \cdot \frac{S_{Glu}}{K_{Gly} + S_{Glu}} \quad \textbf{[S26]}$$

In this eqn. S26, $K_{Gly}$ is $1\cdot 10^{-3}$ M. $q_S^{max}$ has a value of 0.75 mol$_{Glu}$/mol$_{Cx}\cdot$h, as 4 electrons are transferred in the glycolysis process. This rate is equally assumed for all the network reactions, which are considered only kinetically limited by glucose availability. Thus, the kinetics of the

catabolic reactions are modelled according equation S27 ($q_{ri}$, $mol_S/mol_{Cx} \cdot h$), where $\nu_{ri}$ is the stoichiometry factor between the reaction i and the glycolysis ($mol_S/mol_{Glu} \cdot h$).

$$q_{r,i} = f_i \cdot \nu_i \cdot q_{sGly} \quad \text{[S27]}$$

Each substrate consumption yield ($q_{ri}$) is finally multiplied by the biomass present to calculate the reaction rate ($r_i$, $mol_S/L_X \cdot h$).

$$r_i = q_{r,i} \cdot X \cdot \frac{V_r}{V_X} \quad \text{[S28]}$$

## G. Sodium pump kinetics

To equilibrate the intracellular pH, positive charged species (i.e. $Na^+$, $K^+$, etc.) are transported inside/outside the cytoplasm, with a proton transported in opposite direction to maintain the membrane potential. In this model, to control the intracellular pH, we assume the presence of an only sodium pump (Fig. C).

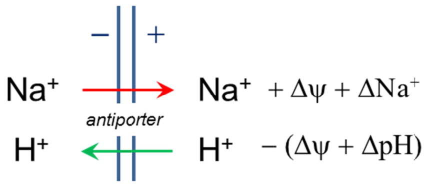


**Figure C** Model of the sodium pump.

As we are not modelling cases where bacteria are in hypotonic or hypertonic environment, we assume that there is not a limitation or excess of external charges concentration, which could imply a significant energy cost or gain. The intracellular and extracellular sodium concentrations in each simulation time step are fixed equal. This simplification is based in the fact that cell is transporting the less costly cation to balance the intracellular pH. This energetic cost is expected minimum reaching a chemical equilibrium between both intra- and extracellular compartments. Moreover, intracellular pH in our model is slightly variable because already all the reactions and transports are pH balanced. Sodium pump is only balancing the pH in the transient states when occurs acids intracellular accumulation or depletion.

Sodium pump is modelled considering a $Na^+/H^+$ antiporter [9]. It acts as a proportional control action to maintain intracellular pH at 7.

$$q_{Na+} = 1 - \exp\left[K_{Na+}\left(10^{-7} - 10^{-pH_{in}}\right)\right] \quad if \quad pH > 7$$
$$q_{Na+} = 0 \quad if \quad pH = 7 \qquad \textbf{[S29]}$$
$$q_{Na+} = \exp\left[K_{Na+}\left(10^{-pH_{in}} - 10^{-7}\right)\right] - 1 \quad if \quad pH < 7$$

$K_{Na+}$ has a value of 100 (dimensionless) to buffer the sodium pump effect.

# H. Transport kinetics across the cell membrane

Both passive and active transports are described in this model. Uncharged species are supposed only passively transported while charged species are actively transported.

*Passive transport*: Passive transport is modelled according to Fick's Law of diffusion, eqn. S30.

$$r_{Diff,i} = Diff_i \cdot \left(S_{i,extra} - S_{i,intra}\right) \cdot X \cdot \frac{V_r}{V_X} \qquad \textbf{[S30]}$$

The $Diff_i$ term in $L/mol_{Cx} \cdot h$ is calculated according eqn. S31.

$$Diff_i = 10^3 \cdot \frac{\mathbf{D}_i \cdot a_x}{\delta_x} \qquad \textbf{[S31]}$$

Where $\mathbf{D}_i$ is the diffusion of the chemical component considered in $m^2/h$, $a_x$ is the superficial area of transport of one mol of biomass $m^2/mol_{Cx}$ and $\delta_x$ is the thickness of the membrane, m.

While the diffusivity parameters ($\mathbf{D}_i$) could be simply estimated according to the measurement of diffusion values in water, $a_x$ values are largely unknown. In our model, the differences in diffusion coefficients are neglected considering that the process is highly regulated by the differences in substrates coefficient partition. This effect is already implemented in the model accurately modelling the species deprotonations concentrations in both intra- and extracellular compartments. Therefore, all the $Diff_i$ coefficients are assumed equal with a value of 100 $L/mol_{Cx} \cdot h$ [10, 11].

*Active transport*: Active transport is carried out by enzymatic control like the intracellular metabolic reactions, thus we assumed a similar kinetic expression for all of them. However, active transport is not thermodynamically controlled as energy input is applied if the transport is endergonic. For this reason, the transport is modelled only with a maximum rate equal than the one for glycolysis, and a Monod-like term referred to the intracellular component transported across the membrane.

$$q_{T,i} = q_{T,i}^{max} \cdot \frac{S_{T,i}}{K_T + S_{T,i}}$$ **[S32]**

$$r_{T,i} = q_{T,i} \cdot X \cdot \frac{V_r}{V_X}$$ **[S33]**

$q_{T,i}^{max}$ is defined like $q_{T,i}^{max} = \nu_i \cdot q_{SGly}$ referred to the reaction that generates the product transported.

Accumulations higher than 10 mM [12] are assumed not physiologically possible so when the intracellular concentrations reach this value, it is assumed that the production is equal to the active transport that reach its maximum value, eqn. S34 ($K_T$ has a value of 20 mM for all the active transports described).

$$r_{T,i} = r_i - r_{Diff,i}$$ **[S34]**

# I. Liquid to gas mass transfer

The dynamics of the components that can be transferred to the gas phase from the liquid should be model. This liquid-to-gas dynamics cannot be neglected as they have approximately the same rate as the biological reactions [13]. The rate of transport from the liquid to the gas depends on an empirical rate coefficient of transference between the gas-liquid phases ($k_La$) that is characteristic of each reactor and of each component considered. Simplifying, $k_La$ in this model is assumed with a value of 15 $h^{-1}$ for $H_2$ and $CO_2$ diffusion to the gas phase. The rate also depends on the difference between the actual concentration in the liquid phase and the equilibrium concentration, calculated through the Henry constant ($K_H$) and the gas partial pressure of the component considered inside the reactor ($p_{gas,i}$), eqn. S35.

$$C_{eq,i} = K_{H,i} \cdot p_{gas,i}$$ **[S35]**

$$r_{L-G,i} = k_L a \cdot \left( C_i - C_{i,eq} \right)$$ **[S36]**

Fixing a constant total pressure inside the reactor, the calculation of the gas flow comes due to the extra pressure produced by the new gas formed.

$$Q_{gas} = \frac{n_{gas-G} \cdot R_g \cdot T}{P - p_{H_2O}}$$ **[S37]**

$n_{gas-G}$ is the total sum of all the moles in gas form generated and $R_g$ the universal ideal gas constant with a value of 0.082 atm·L/mol·K. The extra pressure due to the water vapour ($P_{H2O}$) present in the reactor at the operational temperature is also considered (At 298.15 K, $P_{H2O}$ is $3.12 \cdot 10^{-2}$ atm.).

# J. Cell bioenergetics

Energy is harvested by the cell to use it for maintenance and growth in two ways, via ATP substrate level phosphorylation in the steps of the metabolic network (Fig. A) or to generate proton motive force. Proton motive force is coupled to some catabolic reactions or with the active transports of some substrates.

**Proton motive force generation through catabolic reactions**

It is considered that only the electron transfer between FADH and NADH generates the translocation of one proton. The reaction could proceed backwards if the catabolic process network were thermodynamic favourable in the reverse direction. However, in the conditions of this study (glucose feeding, low $H_2$ partial pressure) only the forward direction is considered. For this reason, we assume that the oxidation of $FADH_2$ in the metabolic network (Fig. A) comes directly linked with the reaction S38 at the same rate (which is simplifying the whole mechanism of eqns. S1—S4).

$$FAD + NADH + H^+ \rightarrow FADH_2 + NAD^+ + \Delta\mu_{H+} \qquad \textbf{[S38]}$$

Then, the concentrations of FAD and $FADH_2$ are constants in the model (same rate of generation and consumption). We fix their concentration according to the value of $\Delta\mu_{H+}$ (which varies with the external pH) making the reaction enough exergonic to translocate one proton across the cell membrane. This imply that in conditions of low pH (big $\Delta\mu_{H+}$), the production of ethanol with one proton translocation is not thermodynamically possible as explained in the main manuscript. In these cases, the possibility of producing ethanol without FAD($H_2$) involved is considered (Fig. A).

**Proton motive force generation through active transports**

Active transports are only considered for products which are all negatively charged. The energy expense for the active transport of an acidic component is calculated according to the model described in the main text (recycling model), equilibrating the ΔpH which implies the movement of an acidic component trough the membrane. Eqn. S39 calculates the total proton motive potential generated/consumed (number of protons intrude or extrude consuming or producing ATP) for active transport produced ($z_i$ relates to the charge of the transported component).

$$y = -\frac{z_i \cdot F \cdot \Delta\Psi + R \cdot T \cdot \ln\frac{[S_{i,in}]}{[S_{i,out}]}}{\Delta\mu_{H^+}} + z_i \qquad \textbf{[S39]}$$

The sodium pump is also an active transport, an antiporter where a proton is translocated in the contrary direction of the sodium molecule (S7 section). The proton pump is modelled according to the recycling model [14] for an antiporter active transport (eqn. S40).

$$y = -\frac{F\cdot\Delta\Psi + R\cdot T\cdot\ln\frac{[Na^{+}_{in}]}{[Na^{+}_{out}]}}{\Delta\mu_{H^+}} + 1 \qquad \textbf{[S40]}$$

Assuming $Na^{+}_{in} = Na^{+}_{out}$ as referred in S7 section, eqn. S40 is simplified, eqn. S41.

$$y = \frac{R\cdot T\cdot\ln\frac{[H^{+}_{in}]}{[H^{+}_{out}]}}{\Delta\mu_{H^+}} \qquad \textbf{[S41]}$$

The number of protons calculated that are translocated per mol of substrate of each reaction is multiplied by the reaction rate. The sum is the total rate of proton translocations ($r_y$).

$$r_y = \sum_{i=1}^{N} y_i \cdot r_i \qquad \textbf{[S42]}$$

The number of protons produced, are simply translated to ATP generated considering the ATP formation rate according eqn. S43. This ATP rate sums to the rate of ATP formed via SLP.

$$r_{ATP} = r_y \cdot \frac{\Delta\mu_{H+}}{\Delta G_{ATP}} \qquad \textbf{[S43]}$$

# K. Anabolism and decay

Eqn. S44 presents the global stoichiometry assumed for anabolism in the model. It is considered that 2 mol of ATP are consumed per mol of biomass formed [15].

$$0.203\cdot Glu + 0.2\cdot NH_3 + 1.72\cdot H_2O + 0.34\cdot NAD^+ + 2\cdot ATP \rightarrow$$

$$\rightarrow 0.22\cdot CO_2 + 2\cdot Pi + 2\cdot ADP + 0.34\cdot NADH + 2.34\cdot H^+ + \mathbf{X} \qquad \textbf{[S44]}$$

For decay, the same eqn. S44 is consider multiplied by -1 but due to the favourable growth conditions imposed in the system studied, the microbial decay impact is almost marginal. For simplicity, the NADH re-oxidation needed for the anabolic process is neglected. It implies an error of no more of ± 20% in the NADH balance of all the simulations presented, which supposes an underestimation of the rate of pathways that occur with NADH oxidation, but in any case affects to the tendencies predicted over the products spectrum with changes in pH.

Anabolism and decay are modelled considering them dependent on the energy available for maintenance and growth and in the anabolic case, limited also by substrates availability. To model the switch between growth and decay dependency on energy availability, factors $f_{ana}$ and $f_d$ are defined.

$$f_{ana} = \frac{\Delta G_{ATP} - 50}{K_{ATP}}; \quad f_d = 0 \quad \textit{if} \quad \Delta G_{ATP} > 50 \text{ kJ/mol}$$
$$f_{ana} = 0; \quad f_d = 0 \quad \textit{if} \quad \Delta G_{ATP} = 50 \text{ kJ/mol} \qquad [\mathbf{S45}]$$
$$f_{ana} = 0; \quad f_d = \frac{50 - \Delta G_{ATP}}{K_{ATP}} \quad \textit{if} \quad \Delta G_{ATP} < 50 \text{ kJ/mol}$$

$K_{ATP}$ has a value of 5kJ·$mol_{Cx}$/$mol_{Glu}$·h to smooth the function discontinuity.

Decay is only defined by the energy limitation but for anabolisim, a glucose limitation termi is included and the anabolism rate is defined like eqn. S46. Ammonium is never a limiting reactant in the conditions simulated (Table A).

$$r_{ana} = f_{ana} \cdot \frac{S_{Glu}}{K_{Gly} + S_{Glu}} \qquad [\mathbf{S46}]$$

The total maintenance energy cost of all biomass in the reactor is calculated as ATP hydrolysis thus it is subtracted to the ATP formation rate (eqn. S47).

$$r_{ATP} = r_y \cdot \frac{\Delta \mu_{H+}}{\Delta G_{ATP}} - m_S^{req} \qquad [\mathbf{S47}]$$

A constant maintenance cost of 4.5 kJ/ $mol_{Cx}$·h is considered [16], so the energy required for the population maintenance ($m_S^{req}$) is calculated in terms of ATP hydrolysis trough eqn. S48.

$$m_S^{req} = \frac{4.5}{\Delta G_{ATP}} \qquad [\mathbf{S48}]$$

# L. Catabolic activity selection

The ATP produced per unit of time is computed for each branch of the metabolic network considering the sum of the ATP produced via SLP plus the ATP produced via proton motive force. In the same way, the NADH oxidized/reduced and $HCO_3^-$ generation/consumption is computed per each metabolic branch.

A MATLAB solver for linear programing problems is used. The inputs of the solver are the total NADH reduced, the total $HCO_3^-$ and the total ATP produced in each catabolic branch defined as starting with glycolysis and ending up in the extrusion of a short carbohydrate out of the cytoplasm (Fig. A).

<u>Example of one catabolic branch:</u>

1 Glucose + 2 $NAD^+$ + 4 ADP + 4 Pi → 2 NADH + 2 $H^+$ + 2 Formate + 2 Acetic acid + 4 ATP

The solver finds, between these catabolic branches a linear combination of $\chi_i$ ($\sum_{i=1}^{N}\chi_i = 1$) that makes cero the production/consumption of NADH (maintaining the redox balance of the cytoplasm) and maximizes the ATP production rate ($mol_{ATP}$/ h). Moreover, $HCO_3^-$, which acts in some reactions as substrate (Fig. A), is included in the optimization because it has to be produced if other catabolic process is consuming it as no $HCO_3^-$ is fed in any simulation case (its production has to be cero or above) (Table A).

$$\chi_i = lin\_opt\left(d\left[\mathrm{NADH}\right]/dt = 0; d\left[\mathrm{HCO_3}^-\right]/dt \geq 0; 0 \leq \chi_i \leq 1\right) \quad \textbf{[S49]}$$

## M. Experimental conditions simulated and cell parameters

The operational conditions simulated in the study are summarised in Table A where only the external pH is modified function of the case analysed.

**Table A** Reactor operational conditions for all simulated case studies

| Variable | Value | Units |
|---|---|---|
| $V_r$ (*Working Volume*) | 2 | L |
| $V_g$ (*Head space*) | 1 | L |
| $P_g$ (*Total gas pressure*) | 1 | atm |
| Q (*Liquid flow*) | 0.25 | L/h |
| HRT (*Hydraulic retention time*) | 8 | h |
| $Glu_{inf}$ | 0.022 | mol/L |
| $NH_4^+$-$Cl^-_{inf}$ | 0.011 | mol/L |

The assumed cell parameters used are summarised in Table B.

**Table B** Parameters assumed for modelling the characteristics of the cell

| Variable | Value | Units |
|---|---|---|
| Internal pH ($pH_{in}$) | 7 | — |
| Membrane potential ($\Delta\psi$) | 0.2 | V |
| Energy of ATP hydrolysis ($\Delta G_{ATP}$) | -50 | kJ/mol |
| Minimum energy necessary for a metabolic reaction to proceed ($\Delta G_{min}$) | -2 | kJ/mol |
| Maximum substrate uptake rate ($q_S^{max}$) | 3 | e-mol/$mol_e \cdot$h |

# References

1. Thauer RK, Jungermann K, Decker K. Energy conservation in chemotrophic anaerobic bacteria. Bacteriol Rev. 1977;41(1):809.

2. Alberty RA. Biochemical Thermodynamics: Applications of Mathematica. Hoboken: Wiley; 2006.

3. Alberty RA. Biochemical thermodynamics and rapid-equilibrium enzyme kinetics. The journal of physical chemistry B. 2010;114:17003-12.

4. Hanselmann KW. Microbial energetics applied to waste repositories. Experientia. 1991;47:645-87.

5. Flamholz A, Noor E, Bar-Even A, Milo R. eQuilibrator--the biochemical thermodynamics calculator. Nucleic Acids Res. 2012;40:D770-D5.

6. Maskow T, von Stockar U. How reliable are thermodynamic feasibility statements of biochemical pathways? Biotechnol Bioeng. 2005;92:223-30.

7. Batstone DJ, Amerlinck Y, Ekama G, Goel R, Grau P, Johnson B, et al. Towards a generalized physicochemical framework. Water Sci Technol. 2012;66(6):1147-61.

8. González-Cabaleiro R, Lema JM, Rodríguez J. Generalised acid-base calculation method for aqueous systems. Proceedings of the 13th IWA World Congress on Anaerobic Digestion Santiago de Compostela2013, June. p. 2012.

9. White D. The physiology and biochemistry of prokaryotes. Third edit ed. New York: Oxford University Press; 2007.

10. Rodríguez J, Kleerebezem R, Lema JM, van Loosdrecht MCM. Modeling product formation in anaerobic mixed culture fermentations. Biotechnol Bioeng. 2006;93(3):592-606.

11. Zhang F, Zhang Y, Chen M, van Loosdrecht MCM, Zeng RJ. A modified metabolic model for mixed culture fermentation with energy conserving electron bifurcation reaction and metabolite transport energy. Biotechnol Bioeng. 2013;110:1884-94.

12. Bar-Even A, Flamholz A, Noor E, Milo R. Thermodynamic constraints shape the structure of carbon fixation pathways. Biochim Biophys Acta. 2012;1817:1646-59.

13. Batstone DJ, Keller J, Angelidaki I, Kalyuzhnyi SV, Pavlostathis SG, Rozzi a, et al. The IWA Anaerobic Digestion Model No 1 (ADM1). Water Sci Technol. 2002;45:65-73.

14. Otto R, Sonnenberg AS, Veldkamp H, Konings WN. Generation of an electrochemical proton gradient in Streptococcus cremoris by lactate efflux. Proc Natl Acad Sci U S A. 1980;77(9):5502-6.

15. Tobajas M, Garcia-calvo E. Determination of biomass yield for growth of Candida utilis on glucose : Black box and metabolic descriptions. World J Microbiol Biotechnol. 1999;15:431-8.

16. Heijnen JJ, Kleerebezem R. Bioenergetics of microbial growth. Encyclopedia of Industrial Biotechnology: Bioprocess, Bioseparation and Cell Technology 2010. p. 1-24.
