# Supplementary material for: Metabolic Energy-Based Modelling Explains Product Yielding in Anaerobic Mixed Culture Fermentations
Source: PLoS One. 2015 May 18;10(5):e0126739. doi: 10.1371/journal.pone.0126739 (PMC4436308; doi:10.1371/journal.pone.0126739)
Supplement: S2 File — (PDF) [file pone.0126739.s002.pdf]

Supporting Information

Metabolic energy-based modelling explains product yielding in anaerobic mixed culture fermentations

R. González-Cabaleiro[1], J. M. Lema[1] and J. Rodríguez[2]

[1] Department of Chemical Engineering, Institute of Technology.
University of Santiago de Compostela, 15782 Santiago de Compostela, Galicia, Spain.
[2]Institute Centre for Water and Environment (iWater) Department of Chemical and Environmental Engineering (CEE)
Masdar Institute of Science and Technology. PO Box 54224 Abu Dhabi, United Arab Emirates

Results for minor products

The results obtained for the prediction of minor products concentrations are not very valuable. They do not permit to obtain final conclusions as the model has not enough precision. We include here the comparison between the results obtained for the concentrations of these products by Temudo et al. 2007 [1] and the results predicted by the model.

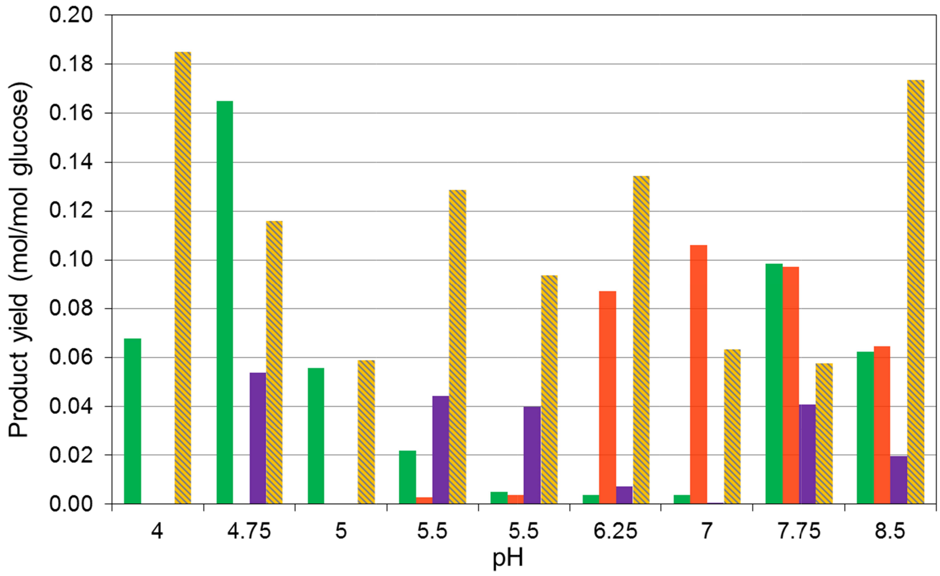

Figure A Experimental steady state yields for minor products as reported by Temudo et al. 2007 [1] (at 20 hours of HRT for pH ≤ 5.5 and at 8 hours of HRT for pH ≥ 5.5). Yields of: ■ lactate; ■ succionate; ■ propionate; ■ glycerol.

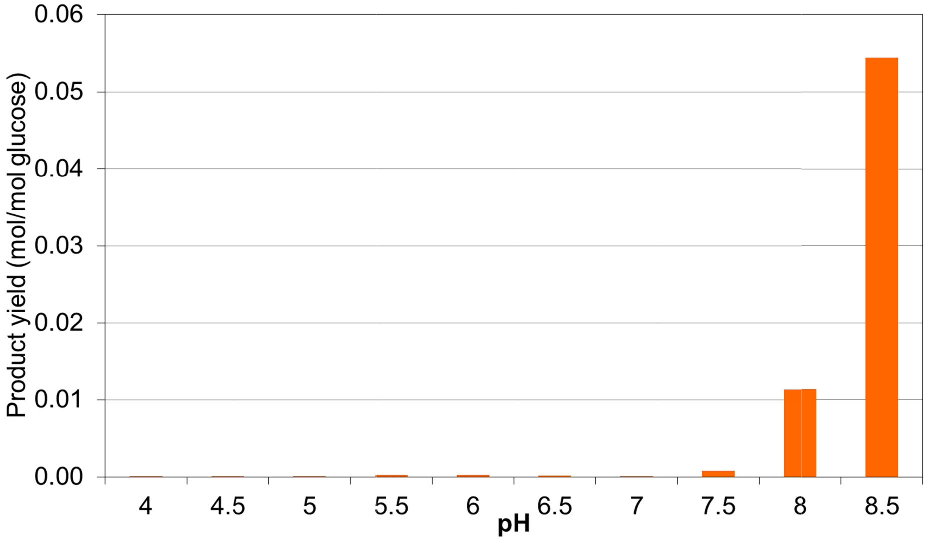

Figure B Model simulation results on the influence of pH on ■ succinate yield.

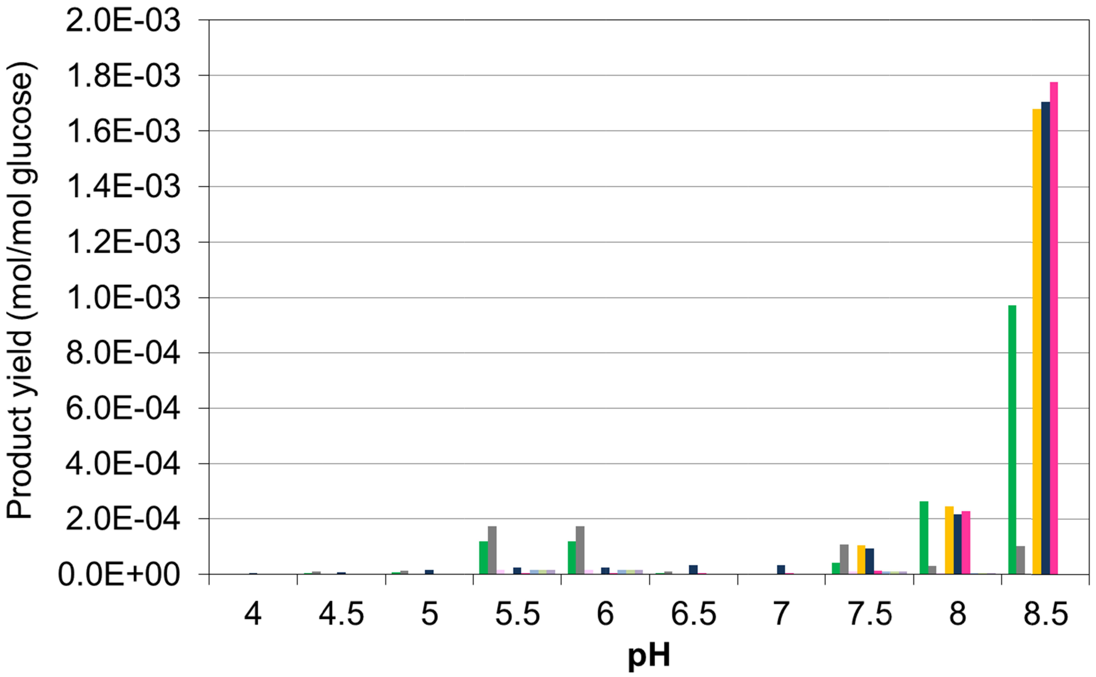

Figure C Model simulation results on the influence of pH on minor products. Yields of: ■ lactate; ■ acetoacetate; ■ acetone; ■ oxaloacetate; ■ malate; ■ fumarate; ■ acetaldehyde; ■ butyraldehide; ■ butanol.

References

1. Temudo MF, Kleerebezem R, van Loosdrecht MCM. Influence of the pH on (open) Mixed Culture Fermentation of Glucose : A Chemostat Study. Biotechnol Bioeng. 2007;98(1):69-79.
